# Carbon Nanomaterial-Based Nanofluids for Direct Thermal Solar Absorption

**DOI:** 10.3390/nano10061199

**Published:** 2020-06-19

**Authors:** Nguyen Trong Tam, Nguyen Viet Phuong, Phan Hong Khoi, Phan Ngoc Minh, Masoud Afrand, Pham Van Trinh, Bui Hung Thang, Gaweł Żyła, Patrice Estellé

**Affiliations:** 1 Institute of Materials Sciences, Vietnam Academy of Science and Technology, 18 Hoang Quoc Viet Street, Cau Giay District, Hanoi 100000, Vietnam; trongtamvatli@gmail.com (N.T.T.); pnminh@vast.ac.vn (P.N.M.); 2 Vietnam Academy of Science and Technology, Graduate University of Science and Technology, 18 Hoang Quoc Viet Street, Cau Giay District, Hanoi 100000, Vietnam; phuongnguyenviet94@gmail.com; 3 Faculty of Basic-Fundamental Sciences, Vietnam Maritime University, 484 Lach Tray Road, Le Chan, Hai Phong 180000, Vietnam; 4 Center for High Technology Development, Vietnam Academy of Science and Technology, 18 Hoang Quoc Viet Street, Cau Giay District, Hanoi 100000, Vietnam; phkhoi@htd.vast.vn; 5 Institute of Research and Development, Duy Tan University, Da Nang 550000, Vietnam; masoudafrand@duytan.edu.vn; 6 Faculty of Electrical—Electronic Engineering, Duy Tan University, Da Nang 550000, Vietnam; 7 Department of Experimental Physics, Rzeszów University of Technology, 35-905 Rzeszow, Poland; 8 Laboratoire de Génie Civil et Génie Mécanique, LGCGM, Université Rennes, 35000 Rennes, France

**Keywords:** carbon nanomaterials, nanofluids, thermal conductivity, viscosity, direct thermal solar absorption

## Abstract

Recently, many scientists have been making remarkable efforts to enhance the efficiency of direct solar thermal absorption collectors that depends on working fluids. There are a number of heat transfer fluids being investigated and developed. Among these fluids, carbon nanomaterial-based nanofluids have become the candidates with the most potential by the heat absorbing and transfer properties of the carbon nanomaterials. This paper provides an overview of the current achievements in preparing and exploiting carbon nanomaterial-based nanofluids to direct thermal solar absorption. In addition, a brief discussion of challenges and recommendations for future work is presented.

## Contents

1. Introduction32. Carbon-Based Nanofluids: Preparation and Stability4 2.1. Carbon Nanomaterials4 2.2. Preparation of Carbon-Based Nanofluids6 2.3. Stability of Carbon-Based Nanofluids6  2.3.1. Use Surface-Functionalized and Sonication Technique for Nanofluid Dispersion and Stability7  2.3.2. Addition of Surfactants83. Properties of Carbon-Based Nanofluids9 3.1. Optical Properties9  3.1.1. Theories for Modeling Optical Properties9  3.1.2. Experiments on Optical Properties13  3.1.3. Photothermal Conversion Performance16 3.2. Thermal Conductivity17  3.2.1. Thermal Conductivity of Graphene-Based Nanofluids17  3.2.2. Thermal Conductivity of CNT-Based Nanofluids20 3.3. Viscosity of Nanofluids Containing Carbon Structures21 3.4. Solar Steam Generation of Nanofluids Containing Carbon Structures22 3.5. Other Properties254. Carbon-Based Nanofluids for Direct Solar Absorption25 4.1. Carbon Nanotube-Based Nanofluids25 4.2. Graphene-Based Nanofluids27 4.3. Hybrid Carbon Nanomaterial-Based Nanofluids28 4.4. Other Carbon Nanomaterial-Based Nanofluids315. Challenges and Recommendations for Future Work32 5.1. Challenges32  5.1.1. Instability of Nanoparticles Dispersion32  5.1.2. High Cost32  5.1.3. Pump Power and Pressure Loss32  5.1.4. Erosion of Components32 5.2. Recommendations for Future Work326. Conclusions33References33

## 1. Introduction

**Figure 1:**
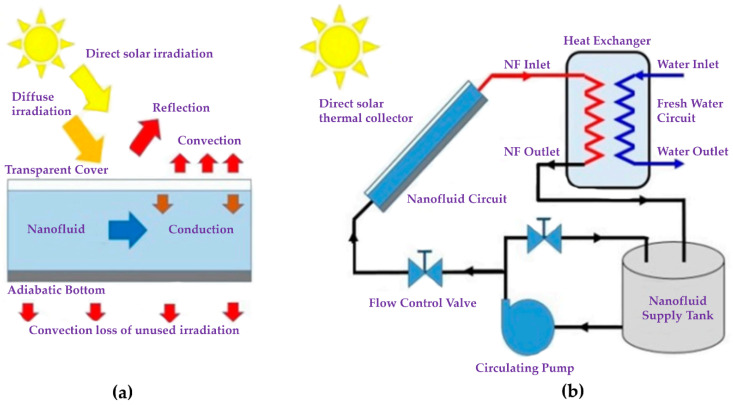
A typical solar thermal absorption collector schematic: (**a**) irradiation and heat loss sources; and (**b**) closed loop system for heat transfer [43] (Reproduced with permission from MDPI).

In recent years, the energy demand of the world has been increasing significantly because of the population explosion and the development of industry. Meanwhile, the limitation and impact on the environment of fossil fuels have posed many challenges in securing energy for the future. To overcome these issues, using clean and renewable energy sources, such as hydropower, geothermal, wind energy, and solar energy, is an optimal solution. Among these energy sources, solar energy could be the best option due to several advantages, such as abundance, inexhaustibility, and cost savings [1,2]. In the most common solar thermal systems being used in various applications, known as the flat-plate black-surface absorbers, solar irradiation is absorbed through a solid surface [3] and converted into the thermal energy by a collector. That energy is then transferred from the collector heat exchanging devices or thermal storage tanks by circulating the working fluid in the collector’s structure. However, the efficiency of these collectors is still poor due to low solar capture, poor heat transfer, and heat losses [4]. These problems are caused by the design and conventional working fluids, such as oil or water-based, with low thermal absorption and heat transfer capacity. Much research has been conducted to enhance the efficiency of solar thermal systems [4,5,6,7,8,9,10,11,12,13,14,15,16,17,18,19,20,21,22,23,24,25,26,27,28,29,30,31,32,33,34,35,36,37,38,39,40,41,42]. To alleviate the above disadvantages, direct absorption solar collectors, which have less heat transfer steps than the common ones, as shown in Figure 1, have been studied and developed [43].

**Figure 2:**
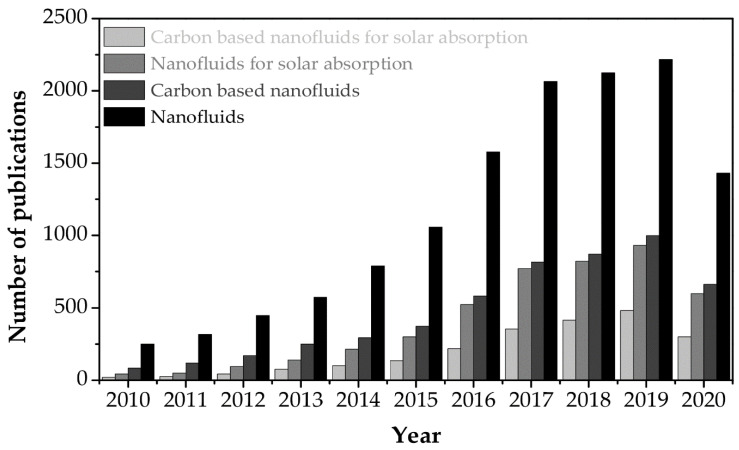
The number of studies on nanofluids for the direct solar thermal absorption (Source: Scopus).

In this type of collector, solar irradiation is directly absorbed and transformed into heat by the working fluid. Because the conventional fluids exhibit poor adsorptive capacity of the solar spectrum range (0.25 µm < λ < 2.5 µm) [7,17], new working fluids with enhanced thermal properties have been prepared in an effort to increase the efficiency of the solar systems. A homogeneous solution of stable suspensions of additives, such as nanomaterials in the base fluid, known as a nanofluid, was first proposed by Choi [44] in 1995. It is considered as a new development step of advanced heat transfer fluids because of the high thermal conductivity and good properties of radiation absorption [45]. Selecting the suitable nanofluids in solar applications is the main factor affecting the performance of the solar systems. Experimental and theoretical results from applications of the variety of both the nanoparticles and the base fluids for the solar systems were reported in numerous studies, as summarized in recent state-of-art review by Qiu et al. [46]. As shown in Figure 2, the number of studies on nanofluids for the direct solar thermal absorption is rapidly rising. The statistical results indicate that nanofluids have attracted intensive attention by their tremendous potential in boosting the efficiency of the direct solar thermal absorption systems. Among the nanoparticles that have been studied, carbon nanomaterials such as graphene (Gr), carbon nanotubes, nanodiamonds and graphite have become the candidates with the most potential because of their outstanding thermal properties. In this paper, recent developments in the preparation and application of carbon nanomaterial-based nanofluids for direct thermal solar absorption are reviewed. Besides, this paper presents existing challenges that need to be overcome and recommendations for future work to achieve the higher efficiency of the solar systems.

## 2. Carbon-Based Nanofluids: Preparation and Stability

### 2.1. Carbon Nanomaterials

**Figure 3:**
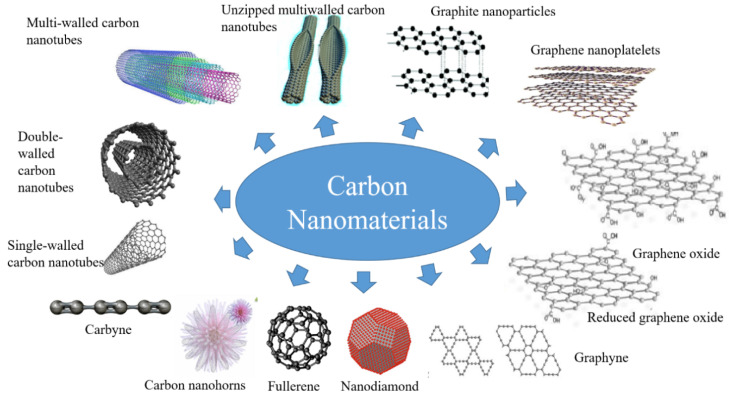
Some allotropes of carbon nanomaterials.

Carbon nanomaterials have many structures, as shown in Figure 3. Due to the unique electronic, optical, thermal, mechanical, and chemical properties, fullerenes (0D), carbon nanotubes (1D), and graphene (2D) carbon nanomaterials are studied by many scientists, providing researchers with the opportunity to make significant progress in basic scientific research and the application of innovative technologies.

Carbon nanotubes (CNTs) have attracted great scientific and technical interest because of their outstanding thermal properties. CNTs have a hybridized sp^2^ bonding that thermal transport can dominate, making them have high thermal conductivity (K) values [47]. At room temperature, Kim et al. [48] used a microfabricated suspended device to measure the thermal conductivity of single-walled carbon nanotubes (SWCNT) over 3000 Wm^−1^ K^−1^. In other work, Pop et al. [49] found the value of K of SWCNT with length 2.6 mm and diameter 1.7 nm nearly equal to 3500 Wm^−1^ K^−1^. On the other hand, Berber et al. [50] observed a thermal conductivity of 6600 Wm^−1^ K^−1^ when they used molecular dynamics simulations and theories.

Graphene has a hexagonal structure, with three strong s bonds in-plane and p orbitals perpendicular to the plane. Graphene layers interact with each other via p bonding out-of-plane [51,52,53]. The heat flow in graphene is anisotropic, the heat flow in-plane is more than 100-fold the out-of-plane directions. At room temperature, the thermal conductivity of graphene about 2000–4000 Wm^−1^ K^−1^ for freely suspended samples [54,55,56]. The acoustic phonons are the main cause of the high-value thermal conductivity of single-layer graphene [57]. When the number of layers is reduced, the Umklapp processes is suppressed, and the absence of crystal defects increases the free path of phonons, resulting in a high K of graphene [58]. The out-of-plane optical (ZO) and the out-of-plane acoustic (ZA) phonon can be grouped in the out-of-plane mode. The longitudinal optical (LO), transverse optical (TO), longitudinal acoustic (LA), and transverse acoustic (TA) phonon can be grouped in-plane mode. Besides, due to the weak van der Waals coupling, the out-of-plane heat flow is limited [59]. Although the in-plane thermal conductivity of graphene is very high for freely suspended samples at room temperature, when graphene is in contact with the substrate or is confined in graphene nanoribbons (GNRs), it is significantly reduced. The K of supported GNRs is about 80 Wm^−1^ K^−1^, that of SiO_2_-encased graphene is about 160 Wm^−1^ K^−1^, and that of graphene supported by SiO_2_ is about 600 Wm^−1^ K^−1^ [60]. This shows that the phonon transmitted in graphene is very sensitive to the surface or edge changes. Due to the coupling and scattering of the phonon with the vibrating modes of the substrate, the thermal conductivity of graphene supported by SiO_2_ is reduced [61]. When the number of graphene layers increases from two to four, the thermal conductivity decreased from 2800 to 1300 Wm^−1^ K^−1^, showing dimensional crossover from two dimensions to bulk by the cross-plane coupling of the low-energy phonons and enhanced Umklapp scatterings [58]. GNRs have phonon scattering with boundaries and edge roughness if the phonon means that the free path is wider than ribbons, therefore the thermal conductivity decrease compares to suspended and SiO_2_-supported graphene [62]. The thermal conductivity of GNRs that are less than 20 nm wide varies sharply with size due to the influence of edge disorders. The thermal conductivity of GNRs as wide as 20 nm is about 1000 Wm^−1^ K^−1^ [63]. Because graphene is atomically thin, it is difficult to experiment with measuring thermal properties. Therefore, modeling and simulation have played an important role in developing an understanding of graphene’s properties. The current simulation methods to understand the heat transfer in graphene as well as predict its thermal properties are molecular dynamics (MD), Boltzmann transport equation (BTE), and non-equilibrium Green’s functions (NEGF) simulations [64,65].

Nanodiamonds have a high specific surface area, tunable surface structure, and exceptional optical and mechanical properties. Due to low phonon scattering and sp^3^ hybridized carbon with short bond length, diamond has a high thermal conductivity—about 2200 Wm^−1^ K^−1^ and as high as 3320 Wm^−1^ K^−1^ in isotropically pure monocrystalline synthetic diamond [64,66]. Carbon nanohorns (CNHs) are closed cages of sp^2^-bonded carbon atoms—they have nanocones, their diameter is typically 2–5 nm, and their length is about 40–50 nm. Their closed cage structure can be considered as fullerenes with a high-aspect-ratio subset. By the oxidation method, they will open and allow access to their inner compartment and increase the surface area. However, due to their elongated shape, their structure is close to that of short single-walled carbon nanotubes and they have similar properties to SWCNT [67].

Nanofluids containing carbon nanomaterials have higher thermal conductivity and heat transfer coefficient than base fluids, even than other nanofluids containing metal and metal oxide nanoparticles, due to the outstanding thermal conductivity of carbon nanomaterials [68]. Compared with other nanomaterials, carbon nanomaterials have a larger surface area, minimal erosion and corrosion, lesser density. In addition, graphene and CNTs have a black surface and a great ability to absorb sunlight. The above properties show that nanocarbon nanomaterials are ideal for the production of nanofluids for direct thermal solar absorption. Therefore, it is necessary to carefully assess the research, achievements and development trends in the use of nanofluids containing carbon nanomaterials for direct thermal solar absorption.

### 2.2. Preparation of Carbon-Based Nanofluids

**Figure 4:**
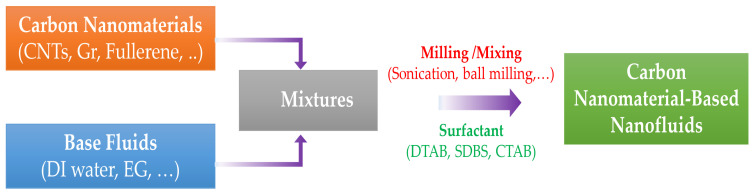
The preparation process of carbon nanomaterial-based nanofluid using the two-step method.

Generally, there are two main methods used to produce the carbon nanomaterial-based nanofluids, including one-step and two-step methods. For the one-step method, carbon nanomaterials are directly prepared in a fluid using several chemical or physical techniques, while the two-step method includes two separate processes: preparing dried carbon nanoparticles and then dispersing them in a base fluid to form a stable suspension. Each method has its advantages and disadvantages. Although the one-step method exhibits good dispersion and stability, the disadvantages including the complexity of the synthesis system and difficulties in controlling the size, shape, and concentration of nanoparticles, making this method less popular in preparing nanofluids. There are only few studies using this method with carbon nanomaterials [69,70]. The two-step method described in Figure 4 is wildly applied to synthesize the carbon nanomaterial-based nanofluids. This method alleviates the disadvantages of the one-step method since the preparation of carbon nanomaterials and nanofluids is dissociated. The size, shape, and concentration of nanoparticles in the nanofluid are controlled more easily. In the process of dispersion, to avoid possible nanoparticle settling that results in poor suspension, it is necessary to employ some techniques such as ultrasonication, mechanical stirring, and surfactants to improve the dispersion and stability of the nanofluids.

### 2.3. Stability of Carbon-Based Nanofluids

Because the stability of the working nanofluids is one of the most important factors for direct solar thermal absorption, it is the starting point of many investigations. Surface-functionalization, sonication techniques and surfactants are used to improve the stability of nanofluids. There are different evaluation methods for the stability of nanofluids, such as spectral analysis, zeta potential, and dynamic light scattering. The main findings in this important topic for carbon-based nanofluids are summarized in this section.

#### 2.3.1. Use Surface-Functionalization and Sonication Technique for Nanofluid Dispersion and Stability

As we know, carbon nanomaterials (CNMs) and graphene are hydrophobic materials with high surface energy, and thus tends to form aggregates in the base fluids. However, functionalizing CNMs considerably improves the wettability of their surface, which allows the CNMs to be dispersed into the fluid, and thus enhances the stability of nanofluids. In ultrasonication, supersonic waves are tasked to separate large clusters into smaller particles. However, there are limitations in sonication due to shorting of CNTs and structure changes occurring to CNMs during extensive sonication.

Hordy et al. [71] studied nanofluids created by suspending plasma-functionalized multiwalled carbon nanotubes (MWCNTs) in denatured alcohol (DA; 85% ethanol, 14% methanol). The stability of the nanofluids were tested over the long term (over a 20-months period) and at a high temperature 65 °C for 1 h, and during evaporation. The curves of transmission spectra for any desired concentration essentially overlapped (less than 1.5% difference in concentration), exhibiting that the nanofluids are extremely stable over time and at elevated temperatures. The stability of nanofluids during evaporation was examined by exposing nanofluids in a fume hood at 20 °C for two cycles of 24 h each. There was more than 50% base fluid evaporated. They concluded that the nanofluids showed higher adsorptive capacity and no agglomeration when their concentration increased.

Similar results were also published by Ivall et al. [72], where the nanofluids were created by mixing oxygen-functionalized multiwalled carbon nanotubes (f-MWCNTs) with deionized (DI) water. They found that the nanofluids maintained stability after over 20 freeze/thaw cycles. After each cycle, sonication was applied to re-disperse clusters formed from the crystallization process. This study offers a quantitative evaluation of f-MWCNT-nanofluid stability as a result of phase change through optical characterization of concentration and particle size. A mapping of the correlation between relative particle size and relative concentration presents all of the data points acquired over all cycles. As with the relative concentration, the relative particle size is calculated in reference to the initial sample prior to phase change cycling. A correlation exists between the two variables. The clustering of nanotubes decreases the surface area of absorbing components, leading to a reduced observed concentration. Relative particle sizes below unity are likely a result of sampling aliquots of the supernatant that contain a higher fraction of relatively smaller particles than the bulk average. The concentration and particle size data suggest that nanofluids made from f-MWCNTs experienced an initial reduction in a number of dispersible components, but the remaining fraction was resilient to phase change cycling after undergoing sonication to disentangle agglomerates and restore nanofluid dispersion.

Li et al. [73] used the β-cyclodextrin (β-CD) modified carbon nanotubes (CD-CNTs) to produce nanofluids for solar energy applications. The ethylene glycol (EG)-based CD-CNTs nanofluids were prepared by the two-step method. Following sedimentation experiments within two months for nanofluids with 0.002 vol%, 0.005 vol%, 0.01 vol%, 0.05 vol% of CD-CNTs, they maintained their stability without any alluviation. The good stability of nanofluids was also confirmed by the zeta potential which was always higher than 50 mV during the testing time. Poinern et al. [74] synthesized carbon nanospheres (CNSs) and dispersed the functionalized CNSs in water. In this work, tetraethylammonium hydroxide ([C_2_H_5_]_4_N[OH]) was employed to functionalize CNS particles with the size of 210 nm. However, the prepared nanofluids showed instability after two or three weeks. The stability of plasma- and acid-functionalized MWCNT dispersions at temperatures up to 150 °C was investigated by Mesgari [75]. Therminol (TH) 55 and propylene glycol (PG) were used as the main solvents. The UV–VIS–NIR spectra of plasma-and acid-functionalized MWCNTs nanofluids with/without heat treatment were compared to investigate their stability. They reported that the destabilization of nanofluids containing acid-functionalized MWCNTs occurred when heating up to 150 °C. However, by using plasma-functionalized MWCNTs, the optical absorption characteristics of the nanofluids (based DI water, TH, EG) remained unchanged with the increase of the temperature up to 150 °C. The obtained results demonstrated that the plasma-induced functional groups on the surface could prevent the agglomeration of MWCNTs in polar and non-polar base fluids at high temperatures.

PUMWNT-based nanofluids for direct absorber solar collectors were prepared by Shende et al. [76]. Functional groups on the surface make PUMWNTs, which were obtained from modified Hummers method, easy to disperse in DI water and EG and kept the suspension stable after two weeks of preparation. The absorption capacity of prepared nanofluids varied linearly as a function of the nanoparticle concentration and obtained the highest absorption at 258 nm. This finding showed the good stability of the nanofluids even at a higher concentration and there was no precipitation and deposition observed at 75 ppm.

Chen et al. [77,78] prepared nanofluids containing graphene oxide (GO) and reduced graphene oxide (rGO) and investigated the dispersion stability under different conditions, including two-months storing and solar irradiation. The obtained results indicated that GO exhibits good dispersion in water due to the existence of rich hydrophilic groups (-OH, COOH) on the nanosheets. There was no presence of precipitations at the bottom of the bottles, pointing out their long-term dispersion stability. The measured absolute zeta potential (ζ) values were higher than 30 mV, indicating the good stability of nanofluids at room temperature. Besides, the absolute ζ values of the nanofluids showed a decrease with the increase of temperature from 30 °C to 70 °C, still to 20 mV. This indicated that nanofluids have good stability from 30 °C to 70 °C, and thus could be considered as suitable working fluids for low-temperature direct absorption solar collectors (DASCs) in solar water heating systems. They also prepared Gr/water nanofluids, but the Gr did not disperse well in water. In other work, Wang et al. [79] also dispersed Gr into heat-transfer oil without any surfactant by high-power and, long ultrasonic short time oscillation. After standing for seven days, nanofluids kept their color and were well dispersed. As shown from the microscopic photo, the graphene nanoparticles were uniformly dispersed in the heat transfer oil. After standing for 30 days, the nanofluids appeared relatively uniform with weak precipitation of nanoparticles.

In the work of Ahmadi et al. [80], pH value was considered as a very important parameter which was associated with the electrostatic charge on the particles’ surface. In order to preserve the stability of nanofluids, the pH should be kept far from the isoelectric point, which is interpreted as a state at the surface of the particle without any net electrical charge (zero zeta potential). They used sodium hydroxide [NaOH] and hydrochloric acid to prepare the samples with various pH. They concluded that the level of acidity of the total working fluid should be changed to pH = 11.6 leading to the most stable mode of graphene nanoplatelets (GNPts)/H2O. A GNPts/water nanofluid has also been investigated for low-temperature direct absorption solar collectors by Vakili et al. [81]. Accordingly, the prepared nanofluids with graphene nanoplatelets had zeta potential value around −31.2 ± 0.5, showing the stability of nanofluids. Unfortunately, visual observations indicated that sedimentation started after 45 days.

#### 2.3.2. Addition of Surfactants

Employing the addition of a surfactant to improve the stability of a nanofluid has been considered as a good method because of its economics and simplicity. Sodium dodecyl sulfate (SDS), sodium dodecyl benzene sulfonate (SDBS), salt, oleic acid, cetyl trimethyl ammonium bromide (CTAB), dodecyl trimethyl ammonium bromide (DTAB), sodium octanoate (SOCT), polyvinyl pyrrolidone (PVP) and Triton X-100 (TX-100) are representative surfactants [82,83,84,85,86,87]. However, the addition of surfactants can induce adverse effects such as foam formation, which is detrimental to heat transfer and fluid flow because it separates the bonding between aqueous solution and nanoparticle.

Choi et al. [82] used a suspension-stability factor ε(t) = (I_0_ − I_t_)/(I_0_ − I_initial_) to estimate the stability of nanofluids containing MWCNTs, where I_t_, I_0_, and I_initial_ are the intensity of the light transmission through the cuvette and the nanofluid as a function of time, the intensity of the light incident to the cuvette, and the initial intensity of light transmission through the nanofluid and cuvette, respectively. They reported that the stabilities of the prepared nanofluids with the addition of SDBS, CTAB, and TX-100 are better than that of nanofluids containing SDS. The SDBS and the TX-100 nanofluids exhibit a better stability compared to others, with the decrease in *ε_t_* less than 1% over a month. The stability of the nanofluids containing SDS reduced significantly, which was nearly 6% at the end of a month-long period. The obtained results demonstrated that the temperature of 85 °C had no effect on the stability of the SDBS, CTAB, and SDS nanofluids, but negatively affected the stability of the TX-100 nanofluids. Choi et al. also conducted a study on the effect of the lower temperature and found that an unexpected result was received for the nanofluids containing CTAB and SDS, as some precipitates formed in the bottom of the bottle at 10 °C [82]. Kim et al. [84] also investigated the effect of the surfactants SDBS, SDS, dodecyl betaine (DB) on the stability of nanofluids containing Gr and CNTs. The obtained results indicated that CNMs nanofluids containing SDBS and SDS surfactants have better stability than that of nanofluids with amphoteric surfactant DB.

## 3. Properties of Carbon-Based Nanofluids

### 3.1. Optical Properties

#### 3.1.1. Theories for Modeling Optical Properties

At present, several theories have been used for modeling optical properties such as the Lambert–Beer approach, the Rayleigh scattering approach, the Maxwell–Garnett theory, the Mie scattering theory and discrete dipole approximation. These theories usually take into account the effect of four parameters, including interparticle distance, particle shape, particle material, and particle size to fundamentally determine the amount of radiation energy that is absorbed or scattered.

##### Lambert–Beer Approach

The spectral transmittance of nanofluids is considered as an important optical parameter for a solar collector which directly effects the efficiency. This parameter can be found from the Lambert–Beer law, in which the intensity of light at a distance *r* can be expressed as the following relation:

(1) $$I(r)=I_{0}e^{-\mu_{ext}r}$$

where *μ_ext_* is the extinction coefficient, which is defined by the sum of the absorption *μ_a_* and scattering *μ_s_* coefficients. L. Mercatelli et al. [88] have suggested the influence rate Φ in a homogeneous medium at a specific distance *r* from a source emitting a unit power by the following relation:

(2) $$\Phi (r)=\frac{3\mu_{s}}{4\pi r}\exp(-r\mu_{eff})$$

where *µ_s_* is the reduced scattering coefficient of the medium and *µ_eff_* is the effective attenuation coefficient. The *μ_eff_* can be determined as the slope of the linear relationship of the natural logarithm of both sides of Equation (2):

(3) $$\ln\left[r\Phi (r)\right]=-\mu_{eff}r+\frac{\ln3\mu_{s}}{4\pi }$$

The absorbed solar energy in the fluids under the no-flow condition, can be obtained from the Equation (4) with the determined extinction coefficients [88]:

(4) $$F(l)=1-\frac{\int_{\lambda_{\min}}^{\lambda_{\max}}I(\lambda )e^{-\mu_{eff}l}d\lambda }{\int_{\lambda_{\min}}^{\lambda_{\max}}I(\lambda )d\lambda }$$

where *I*(*λ*) is the considered Sun spectrum and *l* is path length. *I*(*λ*) is the direct solar radiation, and can be determined by using the blackbody relation [16]:

(5) $$I(\lambda )=\frac{2hc^{2}}{\lambda^{5}}\frac{1}{e^{\frac{hc}{\lambda kT_{solar}}}-1}$$

where *h*, *c* and *k* are the Planck’s constant, the speed of light in vacuum, and the Boltzmann constant, respectively. *T_solar_* is taken as 5800 K.

##### Rayleigh Scattering Approximation

Generally, CNMs considerably decrease the transmittance of the their nanofluids compared to the pure fluid and improve the amount of captured light. This could be due to two issues: (i) the direct absorption of photons by CNMs and (ii) the scattering of light produced by the nanosized particles. The second issue is expected to increase the length of the light path in the fluid, resulting in raising the absorption level. In the case of nanoparticles, which are tested in the wavelength range from 300 to 2300 nm, the Rayleigh scattering regime applies, in which the spectral scattering coefficient varies with the sixth power of the particle size and with the inverse of the fourth power of wavelength [89].

The particle size parameter could be defined as *x = πD/λ*; when *x* << 1, the scattering and absorption efficiency of a spherical particle are determined by using the following relations [89]:

(6) $$Q_{s,\lambda }=\frac{8}{3}x^{4}{\left|\frac{m^{2}-1}{m^{2}+2}\right|}^{2}$$

(7) Qa,λ=4xIm{m2−1m2+2[1+x215(m2−1m2+2)m4+27m2+382m2+3]}

(8) $$Q_{ext}=Q_{s,\lambda }+Q_{a,\lambda }$$

where *m* is the relative complex refractive index of the particles to the fluid. The absorption and scattering efficiencies are used to calculate the extinction coefficient:

(9) $$\mu_{ext,particle}=\frac{3f_{v}(Q_{s,\lambda }+Q_{a,\lambda })}{2D}$$

where *f_v_* is particle volume fraction. For nanoparticles, *x*^4^ << *x*, it is found that *Q_s,λ_* << *Q_a,λ_*. By neglecting the scattering effect, the above Equation (9) could be rewritten as follows:

(10) $$\mu_{ext,particle}=\frac{3f_{v}Q_{a,\lambda }}{2D}$$

In the used equations from Equations (6)–(10), the working medium was considered to be fully transparent. However, in fact, the working medium has a non-negligible absorption coefficient, and thus it can be determined using the following proposed approach:

(11) $$\mu_{ext,base\;fluid}=\frac{4\pi k_{base\;fluid}}{\lambda }$$

The extinction coefficient of the nanofluids can be determined by adding the extinction coefficients of its base fluid and nanoparticles, as expressed in the following equation [89]:

(12) $$\mu_{ext,total}=\mu_{ext,particle}+\mu_{ext,base\;fluid}$$

##### Maxwell–Garnett Approximation

The Maxwell–Garnett theory is a well-known approach for modeling the optical properties of composite systems. This theory has also been used to determine the complex dielectric function of nanofluids with the following equation [89]:

(13) $$\varepsilon_{eff}=\varepsilon_{f}\left[1+\frac{3f_{v}((\varepsilon_{p}-\varepsilon_{f})/(\varepsilon_{p}+2\varepsilon_{f}))}{1-f_{v}((\varepsilon_{p}-\varepsilon_{f})/(\varepsilon_{p}+2\varepsilon_{f}))}\right]=\varepsilon_{1}+\varepsilon_{2}i$$

where *ε_p_*, *ε_f_* are the nanoparticles and base fluid dielectric functions, respectively. The dielectric coefficient is converted to the complex refractive indices by using the following relationships [89]:

(14) $$n_{eff}=\sqrt{\frac{\sqrt{\varepsilon_{1}^{2}+\varepsilon_{2}^{2}+\varepsilon_{1}}}{2}}$$

(15) $$n_{eff}=\sqrt{\frac{\sqrt{\varepsilon_{1}^{2}+\varepsilon_{2}^{2}-\varepsilon_{1}}}{2}}$$

Finally, the extinction coefficient of the nanofluid is estimated by using the following relation:

(16) $$\mu_{ext}=\frac{4\pi k_{eff}}{\lambda }$$

##### Mie and Gans Approach

To overcome the weakness of the Rayleigh scattering model, such as using a larger particle size, Mie’s scattering model has been proposed in order to find the intensity of the scattered radiation. The intensity of Mie scattered radiation is obtained by the summation of an infinite series of terms, rather than by a simple mathematical expression. The scattering and extinction by a particle can be given in terms of scattering and extinction efficiency factors as the following:

(17) $$Q_{s\lambda }=\frac{2}{x^{2}}\sum_{n=1}^{\infty }(2n+1)({\left|a_{n}\right|}^{2}+{\left|b_{n}\right|}^{2})$$

(18) Qeλ=2x2∑n=1∞(2n+1)Re{an+bn}

where *a_n_* and *b_n_* are the Mie scattering coefficients, which are complex functions of *x* given by:

(19) $$a_{n}=\frac{\psi_{n}^{\prime }(mx)\psi_{n}(x)-m\psi_{n}(mx)\psi_{n}^{\prime }(x)}{\psi^{\prime }(mx)\zeta_{n}(x)-m\psi_{n}(mx)\zeta_{n}^{\prime }(x)}$$

(20) $$b_{n}=\frac{m\psi_{n}^{\prime }(mx)\psi_{n}(x)-\psi_{n}(mx)\psi_{n}^{\prime }(x)}{m\psi^{\prime }(mx)\zeta_{n}(x)-\psi_{n}(mx)\zeta_{n}^{\prime }(x)}$$

where *ψ_n_* and *ξ_n_* are known as Riccati–Bessel functions. The calculated extinction efficiency factor *Q_eλ_*, will be employed to determine the extinction coefficient using Equation (10).

##### Discrete Dipole Approximation

The discrete dipole approximation (DDA) is a theoretical basis which was proposed by Draine and then developed as an open source software DDSCAT by Draine and Flatau [90]. This approximation is used to compute the scattering of radiation by particles of arbitrary shape and periodic structures. This method is expected to overcome the weakness of the Maxwell equations, which are only applied for the common shapes, such as spheres, spheroids, or cylinders, so approximate methods are in general required. The DDA does not use any physical approximations but can produce accurate enough results when using a sufficient computer power [91,92,93].

The optical behavior of nanofluids has been studied using these modeling approaches by several researchers, but only few studies predicted the optical behavior of CNT-based nanofluids and compared it to experimental data.

Lee et al. [94] and Hordy et al. [95] measured extinction coefficient and used the Lambert–Beer approach to calculate penetration depth on the absorbed sunlight fraction of nanofluids containing CNTs. The results show that the presence of MWCNTs has a significant influence on the energy storage ability of the nanofluids due to the high solar energy absorption of MWCNTs [95]. Gan et al. [96] used Rayleigh approximations to predict extinction coefficients of ethanol/CNMs nanofluids. The obtained results indicated the good qualitative agreement between the calculated and measured values in the visible range but disagreement in the UV range. This is attributed to the size parameter of nanoparticles and is similar to unity in the visible range and is larger than unity in the UV range. This means the Rayleigh approximation is no longer valid. In this case, the Mie scattering theory should be used to determine the absorption and scattering. It is interesting to notice that when using the Rayleigh approximation, the size parameter defined as *x = πD/λ* should be smaller than one. This means that to satisfy the Rayleigh approximation constraints, the particle diameters must be lower than 159 nm and 318 nm for wavelengths around 0.5 μm and 1 μm, respectively. 

Ladjevardi et al. [97] used the Rayleigh approximation and calculated the extinction coefficients of graphite nanofluids and then compared them to available published experimental data. They investigated the influence of volume fractions and diameter variations of nanoparticles on the extinction coefficient. They reported that the extinction coefficient of nanofluids in UV and visible ranges increases when increasing the volume fraction of nanoparticles. Besides, changing graphite particles diameters have no significant effects for wavelength greater than 1.25 μm, but increase the extinction coefficients, leading to higher energy absorption, for wavelength lower than 1.25 μm. Taylor et al. [7] investigated the extinction coefficient of selected nanofluids and compared the measured results with the Maxwell–Garnett model and Rayleigh scattering approximation model. The obtained results indicated that the Maxwell–Garnett model does not accurately predict the results in short wavelengths (visible range) but accurately predicts in the longer wavelengths. The extinction coefficient of aqueous nanofluids containing MWCNT was investigated by Lee et al. [94]. The experimental data were compared to the calculated results generated from the Maxwell–Garnett model and Rayleigh scattering approximation model. As a result, the conventional Maxwell–Garnett model fails to predict the extinction coefficient of these nanofluids. In contrast, the Rayleigh scattering approximation is able to qualitatively predict the extinction coefficient of water-based MWCNT nanofluid because it takes into account the size effect of MWCNTs. The Mie theory and the DDA method have been recently used by some researchers to estimate the optical properties of non-spherical nanoparticles such as gold nanorods (AuNRs), AgNP, etc. [98,99]. The obtained results indicated that the models do not accurately predict the measured transmittance. To the best of our knowledge, the Mie theory and DDA have not been used for nanofluids containing carbon nanomaterials. Table 1 presents the summary of modeling studies on the optical properties of nanofluids.

#### 3.1.2. Experiments on Optical Properties

**Figure 5:**
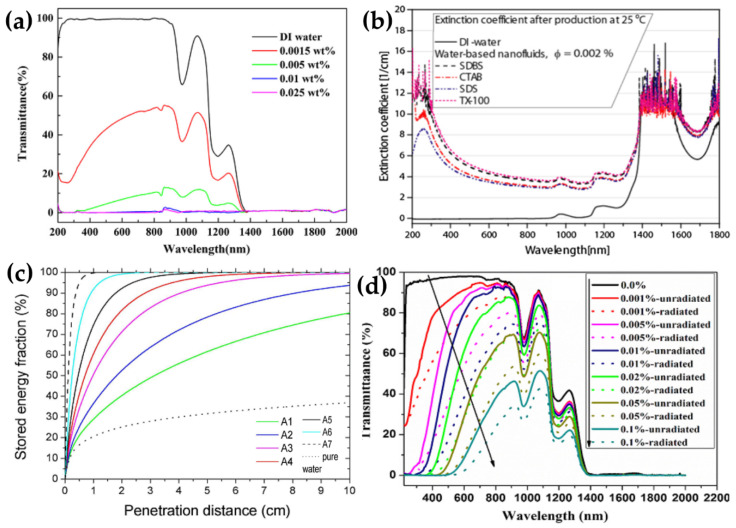
(**a**) Transmittance spectra of DI water and nanofluids containing different MWCNT concentrations [101]; (**b**) the extinction coefficient of water-based nanofluids containing MWCNTs and different surfactants [82]; (**c**) the stored energy fraction versus the penetration distance in the nanofluid with different concentrations (A1 to A7: 0.001 g/L to 0.050 g/L) [37]; (**d**) transmittance spectra of nanofluids containing different GO concentrations before and after being irradiated [77] (Reproduced with permission from Elsevier).

The optical properties of MWCNT/water nanofluid were investigated by Qu et al. [101]. The obtained results indicated that DI water and MWCNT/H_2_O nanofluids have strong absorption bands from 1380 to 2000 nm (Figure 5a). They assumed that DI water can absorb a majority of solar irradiation above 1380 nm for direct solar collection purposes, regardless of the addition of MWCNTs. However, in the lower wavelength range 200–1380 nm, the transmittances of nanofluids containing MWCNT is significantly lower compared to DI water and decreases with increasing the mass fraction of MWCNTs (from 0.0015% to 0.01%). Specially, the nanofluid with 0.01 wt% MWCNT shows nearly zero transmittance in the wavelength range 200–2000 nm. This implies that the nanofluids almost completely absorb in the whole wavelength range. Similar observation was also made by Lee et al. [94]. They concluded that the incident solar energy of a fixed wavelength can be completely absorbed in the penetration depth of 10 cm within nanofluids with extremely low volume fraction of 0.0005 vol% MWCNTs. Hordy et al. [71] found that MWCNT/water nanofluid has an excellent broadband absorber, with a path length (*l*) of 1 cm and at concentrated nanofluid 53 mg/L, as the transmittance was below the detection limit for the spectrophotometer. Choi et al. [82] measured the extinction coefficients of MWCNT/water nanofluids with various surfactants, as shown in Figure 5b. The experimental results indicated that the extinction coefficient of nanofluids is much larger compared to the base fluid at the shorter wavelengths (visible and IR ranges). Besides, the strong dependence of the surfactants on the enhancement of the extinction coefficient was observed. The absorbed sunlight fraction reduces with nanofluids containing TX-100, SDBS, CTAB, and SDS, respectively. From the experimental results, Choi et al. suggested that well-suspended MWCNTs is a key point which plays an important role in improving the extinction coefficient.

Ethylene glycol (EG) with a lower freezing point and higher boiling point compared to DI water is usually used as an anti-freezing agent. Besides, EG can be used to increase the temperature in the case of high temperature solar collectors. Li et al. [73] studied the β-cyclodextrin (β-CD) modified MWCNT (CD-CNTs) dispersed into EG. The transmittance spectra of CD-CNTs nanofluids were measured and the typical spectral peaks were observed for EG absorption at about 920 nm and 1000 nm [41]. The obtained results showed that the average transmittance of nanofluid containing lowest CNT concentration (0.002%) is about 45% lower than that of pure EG. For a light path of 10 mm in the nanofluid, the transmittance is below 5% and zero corresponding to nanofluids containing 0.01 vol% and 0.05 vol% CNT concentration, respectively.

A similar trend was found in PUMWNTs/water and PUMWNTs/EG nanofluids, as observed by Shende et al. [76]. The pure EG and PUMWNTs nanofluids exhibit a perfect absorption from 1400 to 1600 nm. Besides, the transmittance of nanofluids containing PUMWNTs is lower than that of pure EG from 200 to 1400 nm, implying that the solar absorption of base fluid could be enhanced by adding PUMWTs due to the significant increase in extinction coefficient. Other researchers have compared the transmittance spectra of water-based and ethylene glycol-based suspensions [102]. It was found that EG transmittance values are slightly lower than those of water (about 4%) in the UV–Visible range; whereas it was transmitting more than water in the infrared range. However, this trend becomes slightly different by adding the same amount of carbon nanoball (CNB) to the base fluids: the transmittance spectra of aqueous suspensions are higher than EG-based suspensions in approximately the whole of UV–VIS–NIR ranges. On the other hand, water has higher thermal conductivity than EG. However, the advantages of EG with respect to water, which have been discussed above, explain why both of these fluids are considered for solar application.

Sani et al. [37] investigated the optical properties of nanofluids containing single-walled carbon nanohorns (SWCNH) and found that the energy is mainly absorbed in the first layers of fluid. They estimated the fraction of the power stored in the nanofluids with different SWCNH concentrations using Equation (4). Figure 5c shows the calculated stored power distributions along the light propagation direction. For the sample with 0.050 g/L concentration (A7 in Figure 5c), the incident energy is extinct almost 100% in the first centimeter of penetration depth, whereas the sample with 0.001 g/L SWCNT exhibited an energy extinction of about 80% after 10 cm path within the nanofluid, and much higher compared to that of pure water (only 39%). This definitely demonstrates the helpful effect of SWCNH spectral features for efficient solar energy storage.

In other work, Gorji et al. investigated the influence of temperature on optical properties of SWCNT/water nanofluid [103]. When increasing temperature, the nanofluids containing pristine SWCNTs (p-SWCNTs) exhibits a significant decrease in stored energy fraction in comparison with the nanofluid containing carboxyl functionalized SWCNTs (f-SWCNT). This means that the increase of the temperature does not significantly alter the energy storage capability of nanofluids containing f-SWCNTs. Besides, the effect of thermal cycling testing and long term settling on the stored energy fraction of nanofluids were also investigated. In contrast, the reduction in energy storage ability of nanofluid containing f-SWCNTs is much lower, as only 0.5% and 1% decrease in energy storage fraction after thermal cycling testing and long term settling compared to the initial sample.

Similar to CNT nanofluid, Chen et al. [77] found a remarkable improvement in the optical absorption property of the water-based nanofluids containing GO. The obtained results demonstrated that the nanofluids exhibit decreased transmittance in the wavelength range from 220 nm to 2000 nm compared to pure water (Figure 5d). In addition, the transmittance of the GO nanofluids decreased with the increase of GO concentration from 0.001% to 0.1%. This indicated that the optical absorption property of the nanofluids increases as increasing the GO concentration. In addition, the absorption edges of the nanofluids gradually shift to longer wavelengths with higher GO concentrations. This could be originated from the overlapping of the GO nanosheets dispersed into water resulting in the conjugation effect of the π electrons between the overlapped GO nanosheets. Interestingly, it is noted that the transmittance of the nanofluids after being irradiated is lower by about 20–30% than that of the ones before being irradiated.

In addition to the above studies, Sani et al. [104] investigated the light-intensity dependent optical properties of an EG-based nanofluid containing graphite/nanodiamond hybrid suspensions to evaluate their potential for direct absorption solar collectors and solar vapor generation. They have conducted the transmittance measurement of the nanofluids as a function of the input laser fluence for analyzing the optical limiting performances at three wavelengths in the UV, visible and near infrared range. The obtained results indicated that vapor bubbles were generated around the nanoparticles at light intensities comparable to sunlight concentration systems. This demonstrated that the graphite/nanodiamond hybrid material could be considered as a promising potential candidate for solar vapor generation systems. Table 2 presents summary of experimental results on optical properties of nanofluids.

#### 3.1.3. Photothermal Conversion Performance

In a DASC, the working fluid converts the energy of incoming radiation into heat (photothermal conversion). After absorbing radiation, the temperature of fluid will increase. The temperature rise of fluid depends on the thermal properties as well as the optical properties of the fluid. It is obvious that adding CNMs to the based fluid will improve the photothermal properties. Qu et al. [101] evaluated the photothermal properties of nanofluid containing MWCNT. The temperature of the nanofluid increases faster than DI water in the same lighting time of 45 min. After 45 min lighting time, the temperature increase of MWCNT/water nanofluid (0.005 wt% concentration) is 10.0 °C (or 15.2%) higher than the temperature increase of DI water. They found that the optimal MWCNT concentration is 0.01 wt%. The increased heat loss of the fluid with increasing MWCNT concentration may be due to more infrared emission. With large concentrations of MWCNT, it is possible that only the upper fluid layer absorbs light, so the temperature of the lower fluid layer is smaller than the optimal concentration liquid. Similar observations were found in the case of GO/water nanofluids by Chen et al. [77]. With the same lighting time of 7500 s, GO/water nanofluid with concentration of GO from 0.001% to 0.1% has a higher temperature rise than DI water, nanofluid with a GO concentration of 0.02% have the largest temperature increase. Therefore, in DASC, to achieve the highest efficiency, it is necessary to select the optimal of CNMs concentration for the working fluid. Chen et al. [78] also compared the photothermal conversion of three types of nanofluids: rGO/water, GO/water and Gr/water. With a lighting time of 9000 s, the temperature increase of all three nanofluids is higher than that of DI water. This demonstrates that CNM has improved the photothermal conversion of nanofluids. Among the three types of nanofluids, they found that the rGO/water nanofluid had the best photothermal properties. The rGO/water nanofluid has a higher thermal conductivity than Gr/water, absorbing more radiation than Gr/water, so photothermal conversion of rGO/water is better than of Gr/water. However, of the three types of nanofluids, although the Gr/water nanofluid has the highest thermal conductivity and the best optical absorption, its photothermal conversion was the lowest. This could be due to the fact that when the temperature is high, the Gr nanosheets are agglomerated, reducing the stability of the nanofluid, thus reducing photothermal conversion. The requirements of working fluids in DASCs are good dispersion as well as high optical absorption property.

### 3.2. Thermal Conductivity

Due to the high thermal properties, researchers have utilized carbon nanomaterials as nanoparticles in preparing nanofluids. Many results of the thermal conductivity enhancement of nanofluids containing carbon nanomaterials have been previously reported [106,107,108,109,110,111,112,113,114,115,116,117,118,119,120,121,122,123,124,125,126,127,128,129,130,131,132,133,134,135,136,137,138,139,140,141,142,143,144]. In this work, we review remarkable studies and provide summaries for two main types of carbon nanomaterials namely graphene and CNTs.

#### 3.2.1. Thermal Conductivity of Graphene-Based Nanofluids

Graphene with a measured thermal conductivity in the range from 3000 to 5000 W/mK at room temperature is considered as a material added to increase the thermal conductivity of the base fluid.

**Figure 6:**
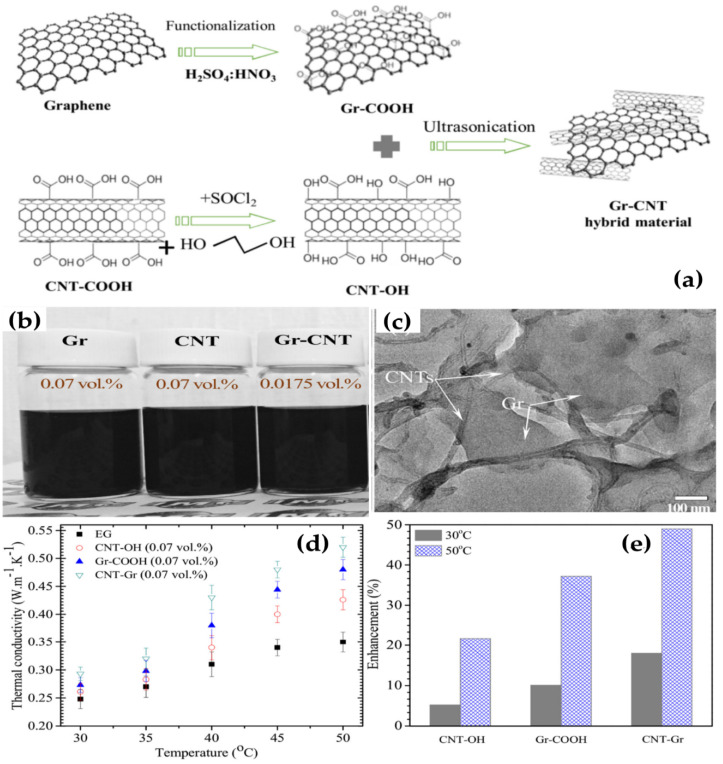
(**a**) Schematic view of the preparation process of Gr-CNT hybrid material; (**b**) optical images of nanofluids; (**c**) transmission electron microscopy (TEM) image Gr-CNT hybrid material; (**d**) thermal conductivity and (**e**) thermal conductivity enhancements of nanofluids containing carbon nanomaterials [116] (Reproduced with permission from Elsevier).

Yu et al. [106] reported that the thermal conductivity of EG was increased up to 86% by the introduction of graphene at 5 vol%. Shende and Sundara [107], Baby et al. [108] examined the effect of base fluids on thermal conductivity by comparing two nanofluids created by dispersing graphene in water and EG without any surfactant. The results indicated that the thermal conductivity enhancement of Gr/EG nanofluid was remarkably lower than that of the Gr/water nanofluid at the same volume fractions. Alicia et al. [109] employed oil as the base fluid to prepared graphene nanoparticle-based nanofluids. The thermal conductivity was significantly improved from 2.6% to 69.31% as the temperature increased from 20 °C to 100 °C, for 0.01–0.1 wt% of graphite nanoparticles (GNPs). Kim et al. [110] examined the dependence of the thermal conductivity on the graphene particle size and concluded that decreasing the average particle size would result in an increase of thermal conductivity. Abdulla et al. [111], Ghozatloo et al. [112], and Gandhi et al. [113] investigated nanofluids prepared by dispersing the functionalized graphene in water without any surfactants and found that the thermal conductivity was substantially enhanced even at a lower concentration. In addition, the enhancement improved with increasing the graphene concentration and depended on the temperature. Ahammed et al. [114] studied the thermal conductivity of Gr/H_2_O nanofluids in the temperature range from 10 °C to 50 °C. An interesting observation from this study was that the average thermal conductivity enhancement percentage with the increase in volume concentration (say from 0.05% to 0.15%) was found to be 3.3% higher when compared with that of the average enhancement with the increase in temperature from 10 °C to 50 °C. Vallejo et al. [115] reported the thermal conductivity of PG water mixture-based nanofluids with mass fractions of PG nanoparticles up to 0.01. Experiments were conducted at various temperatures from 293.15 K to 323.15 K. They found that thermal conductivity was considerably boosted when the PG concentration increased, from 4.7% enhancement with 0.25 wt% PG up to 16% enhancement for 1.0 wt%. Trinh et al. [116] used Gr-GNT hybrid materials for preparing nanofluids and found that the thermal conductivity increases up to 49% compared to based nanofluids and nanofluid containing Gr or CNTs (Figure 6). A general comparison about the thermal conductivity of other nanofluids containing graphene is given in the Table 3.

#### 3.2.2. Thermal Conductivity of CNT-Based Nanofluids

The experimental results of recent studies have indicated that CNTs are potential candidates for enhancing the thermal properties of the base fluid. Estellé et al. [129] investigated the effect of nanoparticle volume fraction, temperature, carbon nanotube aspect ratio, and different kinds of surfactant (SDBS, Lignin, Sodium polycarboxylate) on the thermal conductivity enhancement of nanofluids. Talaei et al. [130] investigated the effect of functional group concentration induced on the surface of MWCNTs on the thermal conductivity of nanofluids. They reported that the increase of the functional group concentration will help to improve the stability and thermal conductivity of the prepared nanofluids. Nanda et al. [131] investigated the effects of the base fluid, poly-alpha-olefins (PAO) and EG on the thermal conductivity of the MWCNTs nanofluid and found that the thermal conductivity enhancement of the EG-based fluid (40%) was higher than that of the PAO-based fluid (33%) with the MWCNTs concentration of 0.03 vol%. Aravind et al. [132] also studied this factor with water and EG. The result showed that the thermal conductivity enhancement of the EG-based fluid was much higher than that of the PAO-based fluid, 35% and 12% respectively. Chen and Xie [133] studied the contribution of various carbon nanotube structures, including single-walled, double-walled, few-walled and multiwalled carbon nanotubes, to the performance of nanofluids. They found that at the same temperature (55 °C) and CNT concentration (0.2%), SWCNTs, double-walled carbon nanotubes (DWCNTs) and MWCNTs nanofluids showed 15.6%, 14.2%, and 12.1%, respectively, in thermal conductivity enhancement. Nasiri et al. [111] conducted the same experiment with 0.25 wt% of each CNT structure. The results indicated that CNT structures with smaller diameter show greater enhancement in thermal conductivity. Hence, SWCNT-based nanofluids had the highest thermal conductivity enhancement among the investigated nanofluids. For comparative purposes, more results on recent thermal conductivity measurements of CNT-based nanofluids can be found in Table 4.

### 3.3. Viscosity of Nanofluids Containing Carbon Structures

The rheological behavior of nanofluids is diverse, as presented in recent review papers in this field [145,146,147,148]. From the application point of view, nanofluids considered as potential heat transfer fluids should preferably exhibit low viscosity and Newtonian behavior. However, experimental studies on this issue show that nanofluids containing carbon structures present complex rheological behavior in some cases. Vallejo et al. showed the Newtonian behavior of nanofluids containing functionalized graphene nanoplatelet with a mixture of propylene glycol and water as a base fluid [115,143].

Halelfadl et al. [149] investigated the viscosity of water-based nanofluids containing CNT in mass concentrations up to 0.75%. They concluded that nanosuspensions containing low fraction of nanotubes exhibit Newtonian nature, and above a volume fraction of 0.055% it changes to non-Newtonian shear-thinning. The shear-thinning characteristic of nanofluids containing MWCNT has been reported by Phuoc et al. [150]. They prepared nanofluids in mass concentrations up to 3%. Again, nanosuspensions of low fraction of particles could be consider as Newtonian. Garg et al. [142] showed that ultrasonication time has an influence on the viscosity of deionized water-based nanofluids containing 1 wt% of MWCNT. They showed that no matter how long the ultrasonic action was performed, those type of nanofluids exhibited non-Newtonian nature, while deviations between the viscosity values of samples prepared with various sonication times could be observed. The same trend has been described by Sadri et al. [128].

Recently, nanofluids containing nanodiamonds appear as an interesting material for advanced heat applications. However, those materials exhibit non-Newtonian behavior, as presented by Minakov et al. [151,152]. They examined the viscosity of water-based nanofluids containing nanodiamonds (ND) with an average size of 5 nm. The shear-thinning behavior of ethylene glycol-based nanofluids containing ND and ND/graphite mixture was reported by Żyła et al. in [153] and [154] respectively. A comprehensive study on the thermophysical properties of nanofluids containing two types of nanodiamonds with different purities (97% and 87%) was performed in [153]. Beside the rheological properties, the thermal conductivity, isobaric heat capacity, mass density and dielectric profile of those materials were presented. From the rheological point of view, those nanofluids exhibit interesting properties. Viscoelastic and thixotropic (which means that the viscosity, and the shear stress, depend on the time of shear and sample history, as defined in [154]) structures have been observed there. A viscoelastic structure could be also observed in ethylene glycol-based nanofluids containing a mixture of ND and graphite, as presented in [155]. In both nanofluids described there, the flow behavior could be modelled with the Herschel–Bulkley (HB) model.

The viscosity of ethylene glycol-based nanofluids containing carbon nanohorns was investigated by Salevam et al. [156]. They showed that nanosuspensions of low volume concentrations (up to 0.25%) exhibit Newtonian nature, while higher concentration induces non-Newtonian behavior.

According to the available experimental data, one could not clearly classify the nanofluids containing carbon structures as Newtonian or non-Newtonian fluid. Despite this, the concentrations of nanoparticles in nanofluids used in solar applications are usually quite low; before considering the use of particular carbon-based nanofluid, its rheological behavior and viscosity should be experimentally evaluated, specifically at temperatures relevant for solar applications. Summary of some results on experimental studies on viscosity of nanofluids containing carbon nanoparticles has been presented in Table 5.

### 3.4. Solar Steam Generation of Nanofluids Containing Carbon Structures

Solar steam generation has been considered as a highly efficient photothermal conversion method with a wide range of applications, including water purification, distillation, power plants, and seawater desalination. It is well-known that the use of traditional working media will result in low steam generation efficiency for solar steam generation. Recently, many studies have sought to find new nanomaterials with an excellent light absorption performance to improve the photothermal conversion efficiency of solar devices [157,158,159,160,161,162,163]. For example, Wang et al. [164] reported fast and efficient evaporation could be obtained when using a bio-inspired gold nanoparticle surface evaporation approach through the localized plasmonic heating. Fang et al. [165] proposed a method for the direct and quantitative analysis of light-induced steam generation by nanoparticles from the nanoscale to macroscale. Jin et al. [166] performed both steam generation experiments and mathematical modeling to investigate the different concentrations of gold nanoparticles dispersions under focused natural sunlight of 220 Suns. The obtained results indicated that the initial stage of steam generation is mainly caused by localized boiling and vaporization in the superheated region resulted from highly non-uniform temperature and radiation energy distribution. 

**Figure 7:**
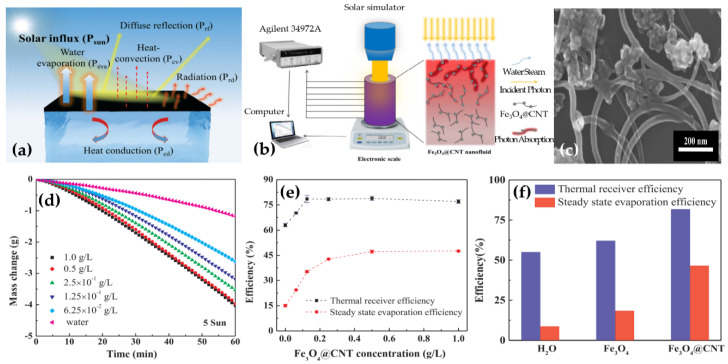
(**a**) Energy balance diagram of a solar water evaporation system [162]; (**b**) experimental setup for solar steam generation; (**c**) SEM images of Fe_3_O_4_@CNT hybrid materials; (**d**) evaporation mass change curves; (**e**) thermal receiver efficiency and steady-state evaporation efficiency; and (**f**) thermal receiver efficiency and steady-state evaporation efficiency of Fe_3_O_4_ and Fe_3_O_4_@CNT nanofluids [168] (Reproduced with permission from Elsevier).

Carbon-based materials such as carbon black, CNTs, graphene, GO, rGO, etc. have received great attention for nanofluids in the photothermal applications due to their broad light absorption, high stability, lightweight and low cost. Ghafurian et al. [167] reported that nanofluids containing SWCNTs, MWCNT-OH and MWCNTs-COOH could improve the evaporation efficiency in which nanofluid containing MWCNT-OH exhibited the most promising optical and stability properties, with higher evaporation rates as compared to the other nanoparticles. Shi et al. [168] employed magnetic Fe_3_O_4_ decorated CNTs (Fe_3_O_4_@CNT) for separation from water under the action of a magnetic force. The nanofluid containing 0.5 g/L Fe_3_O_4_@CNT showed a high evaporation efficiency up to 60.3% under a solar illumination power of 10 sun (1 sun = 1 kW·m^−^^2^), and a thermal receiver efficiency of 88.7% under a solar illumination power of 1 sun (Figure 7). The obtained results proposed the prepared systems as an approach to significantly reduce the material consumption in the design of solar evaporators and to realize broad solar energy applications including seawater desalination, vapor generation, etc.

**Figure 8:**
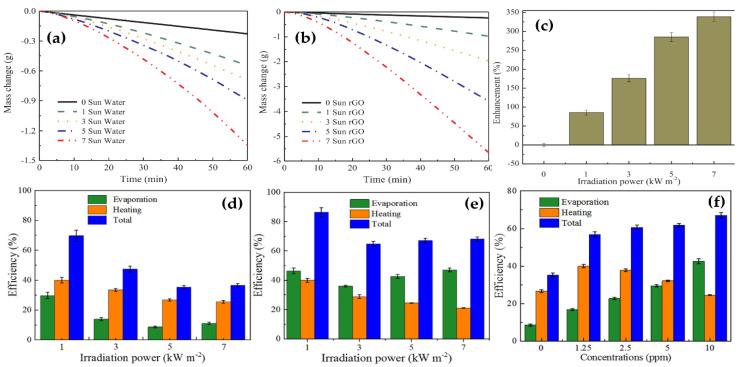
The evaporation amount of (**a**) water and (**b**) the nanofluid containing rGO versus time under different light intensities; (**c**) the enhancement effect of the nanofluid in comparison with water, the evaporation, heating; and total thermal efficiency of (**d**) water and (**e**) nanofluid; and (**f**) the evaporation, heating, and total thermal efficiency of the nanofluid versus different concentrations [170] (Reproduced with permission from Elsevier).

Ni et al. [169] worked on nanofluids containing graphitized carbon black, carbon black, and graphene. The vapor generation efficiencies were reached up to 69% at solar concentrations of 10 sun which significantly improve in both transient and steady-state performance compared to other reports. By using numerical and analytical heat transfer models, it was suggested that nanofluid heating and vapor generation is caused by classical global heating of the suspension fluid. Similarly, Liu et al. [170] reported that highly efficient volumetric solar steam generation could be obtained when using rGO nanofluids with good stability and light absorption capability. The obtained results demonstrated that the improvement in evaporation efficiency has resulted from a hot area formed at the water-air interface and the unique lamellar structure of rGO leading to sunlight absorbed by the hot area to generate steam locally. Figure 8 shows the evaporation efficiency, heating efficiency, and total energy utilization efficiency. As a result, the evaporation efficiency increases with the increase of rGO concentration. The sum of the evaporation and heating efficiencies was above 50%, higher compared to pure water (35%), implying the enhancement in solar steam generation. Table 6 presents selected experimental results on the solar steam generation of carbon nanomaterial-based nanofluids.

### 3.5. Other Properties

In addition to the abovementioned thermal conductivity and viscosity, other factors also have an influence on the potential applicability of nanofluids. One might find comprehensive review papers on other properties of nanofluids, such as surface tension [176], isobaric heat capacity [177,178,179], entropy [180], pool boiling [181], electrical conductivity [182,183], and electric breakdown voltage [184].

## 4. Carbon-Based Nanofluids for Direct Solar Absorption

### 4.1. Carbon Nanotube-Based Nanofluids

Recently, the application of carbon nanotube-based nanofluids for direct thermal solar absorption has become attractive. Many studies have been conducted and show the potential of carbon nanotube-based nanofluids in this field.

Hordy et al. [95] demonstrated the absorptive solar spectrum capacity of carbon nanotube-based nanofluids by quantitatively examining the long-term, high-temperature stability, and optical properties of CNT nanofluids for use in direct solar absorption. Four base fluids, namely water, ethylene glycol, propylene glycol, and Therminol^®^ VP-1, are employed to prepare the nanofluids. They reported that the glycol-based nanofluids exhibited long-term stability at room temperature for a period up to 8 months, when the based fluid was water, the CNTs were sediment. When the based fluid was non-polar Therminol^®^ VP-1, the CNTs agglomerated. At temperature as high as 175 °C, glycol-based nanofluids do not show the agglomeration of the CNTs. An optical spectrum of nanofluids containing CNTs showed that it was capable of absorbing most of sunlight, nearly 100% of solar energy at 10 cm of fluid thickness, even at low concentrations and small collection volumes. They concluded that the glycol-based nanofluids with the excellent absorption properties, as well as the good long-term and high-temperature stability, were ideal candidates for direct solar thermal energy absorption.

Khullar et al. [185] studied the heat transfer mechanism in a volumetric absorption system using two types of nanofluids (ethylene glycol containing amorphous carbon nanoparticles and distilled water containing MWCNT) as the working fluids. Under low radiation flux conditions, they compared the surface absorption system using copper-coated TiNOX^®^ as the absorbing surface to the volume absorption system. They found that MWCNT nanofluids showed higher stagnation temperatures than amorphous carbon nanoparticles suspensions. In addition, the volumetric absorption system converts solar energy into thermal energy in working fluids more efficiently.

Karami et al. [186] used functionalized CNTs in water nanofluids for the direct absorption solar collectors (DASCs). Seven samples of nanofluids with different volume fractions were prepared based on the two-step method, concentration of samples S1, S2, S3, S4, S5, S6, S7 are 0, 5, 10, 25, 50, 100, 150 ppm, respectively. They found that optical properties and high thermal conductivity varied with the concentration of functionalized carbon nanotubes (f-CNTs). Transmission spectra show that f-CNT has a great influence on the ability to absorb light and just a small amount of f-CNT also reduces the transmittance. By adding 150 ppm of f-CNTs to the pure water, the extinction coefficient and thermal conductivity enhancement of the nanofluid was significantly increased from about 4.1 cm^−1^ to 32.2%. The thermal conductivity of nanofluids increase with f-CNT concentration as well as with temperature. In their opinion, the overall efficiency of DASC will increase if the f-CNTs nanofluids is used as working fluids.

Gorji et al. [103] reported on the optical characterization of pristine SWCNTs aqueous suspensions nanofluids compared to that of chemically functionalized SWCNTs/water. The results showed that the absorption spectra of the nanofluids was significantly improved by the presence of SWCNT nanoparticles. However, the absorption reached a maximum value at a certain concentration of SWCNTs and decreased by an increase in sample concentration due to both agglomeration and self-absorption phenomena. According to their study, the concentration of 30 mg/L and 60 mg/L for pristine and carboxyl functionalized SWCNTs nanofluids, respectively, gained the highest spectral absorption. The extinction coefficient of nanofluids containing SWCNT were measured and compared at temperatures ranging from 25 to 90 °C to investigate the dependence of optical properties on temperature. They found that increasing the temperature reduced the absorbance of nanofluid. At high temperatures, it is possible that the number of collisions between nanoparticles increases, which increases the agglomeration and settling out of suspension, leading to reduced particle density. However, due to the better stability of the nanofluid, the optical properties of nanofluids containing functionalized SWCNT depend on temperature less than nanofluids containing pristine ones. In addition, when they undergo a thermal cycle, after long time (three months) the results indicated that functionalized SWCNT nanofluids had much better stability and energy storage ability than pristine ones.

Lee and Jang [187] considered aqueous MWCNT suspensions to analytically investigate the efficiency and temperature fields of a nanofluid volumetric receiver (NVR). The extinction coefficients of MWCNT/water nanofluids to the wavelength from 200 to 2000 nm were experimentally measured by a UV–Vis–NIR spectrophotometer. The obtained results showed that the NVR efficiency was dependent on the Nusselt number of heat loss, the nanoparticle concentration, the Peclet number, and the collector aspect ratio.

Delfani et al. [100] employed CNTs to synthesize nanofluids as working fluids for the direct absorption solar collectors. They experimentally investigated the performance characteristics of CNT-based nanofluids and compared them with the results obtained from the numerical model. They predicted the output temperature parameters by combining the energy equation with the radiative heat transfer equation and changing the input parameters. The carboxyl functionalized multiwalled carbon nanotubes disperse in water and ethylene glycol. According to their report, these nanofluids exhibited a long-term stability (no settling was detected after a month). They found that increased CNT concentration and temperature led to an increase in the conductivity of the nanofluid, the ability of heat transfers to increase, and an enhancement in collector efficiency. The collector was tested for different inlet temperatures between 30 and 50 °C and the flow rates were 54, 72, and 90 L/h. The results indicated that the collector efficiency was improved by increasing nanofluid volume fraction and flow rates. The authors pointed out that the lower flow rate will cause the fluid temperature to rise further due to the high fluid residence time. The maximum efficiency of the collector was achieved at the flow rate of 90 L/h and when the concentration of CNT was 100 ppm. Compared with using base fluid as a working fluid, the efficiency of the collector using nanofluid increases by 29% at the same flow rate.

Shende and Ramaprabhu [76] theoretically and experimentally studied partially unzipped multiwalled carbon nanotube (PUMWCNT)-based nanofluids consisting of PUMWCNTs dispersed in DI water and ethylene glycol for direct absorption solar thermal energy systems. PUMWCNTs were created by unraveling the outer few layers of multiwalled carbon nanotubes without affecting the inner core walls. The nanofluids exhibited the long-term stability (up to two months). The study showed that the extinction coefficient of nanofluids was significantly increased by adding PUMWCNTs into the base fluids. Thermal conductivity enhancements of 27% and 21% were achieved for DI water and ethylene glycol-based nanofluids, respectively, at 50 °C for 0.03 vol%. This results confirmed that PUMWNT-dispersed nanofluids were promising to increase the overall efficiency of direct absorber solar collectors.

In the experimental study of Kasaeian et al. [188], two types of nanoparticles consisting of MWCNT and nanosilica dispersed in ethylene glycol were used and compared to investigate the efficiency of a solar direct absorption parabolic through collector. The nanosilica were used in this study due to their high thermal conductivity. They reported that the highest temperature difference between outlet and inlet was up to 32.5 °C with a 0.3% MWCNT/EG nanofluid. This value was 15.8 °C higher than that using the base fluid. The thermal efficiency of fluids containing MWCNTs, nanosilica, and ethylene glycol was 72.8%, 63.6%, and 55.8%, respectively. This meant MWCNT nanofluids had a greater thermal efficiency than ethylene glycol and nanosilica nanofluids with the same concentration. Tam et al. [189] investigated the thermal conductivity and photothermal conversion performance of ethylene glycol-based nanofluids containing MWCNT. They reported that the highest photothermal conversion was obtained about 4.2% after a 30 min lighting with nanofluid containing 0.48 vol% CNT concentration compared to pure EG.

### 4.2. Graphene-Based Nanofluids

Due to the great enhanced thermal-optical properties of nanofluids consisting of graphene, many researchers employed graphene-based nanofluids as working fluids to evaluate the efficiency of direct thermal solar absorption.

Chen et al. [78] prepared reduced graphene oxide/water nanofluids to use in low-temperature DASCs. The nanofluids showed excellent thermal stability without any visible precipitations for 2 months. The photothermal conversion performance of the RGO/water nanofluid was evaluated and compared to that of water and the GO/water and GE/water ones in the same experimental condition. The experimental results showed that the RGO/water nanofluid could reach the highest temperature, up to 82 °C, while that of the GO/water and the GE/water nanofluids were only 77 °C and 75.5 °C, respectively. In addition, 96.93% energy of sunlight was converted to thermal energy by RGO/water nanofluid at 30 °C. This value was also higher than that of the other nanofluids in the research. Therefore, they concluded that reduced graphene oxide/water nanofluids with excellent performance could be a good working-fluid candidate in low-temperature DASCs.

Liu et al. [190] analytically and experimentally investigated the efficiency of high temperature direct solar collectors and concentrated solar collectors using graphene/ionic liquid nanofluids as the absorbers. They dispersed different fraction of graphene into [HMIM]BF_4_ without any surfactants. With 0.0005 wt% of graphene in the working solution, they obtained more than 40% decrease of the average transmittance in the 300–850 nm light range. Moreover, 100% sunlight was wholly absorbed when graphene concentration increased to 0.01 wt%. The temperature also varied as a function of the graphene fraction. This parameter raised to 650 K at 0.002 wt%, then fell to 630 K at 0.01 wt% graphene concentration. The receiver efficiency of graphene/[HMIM]BF_4_ also decreased from 0.72 to 0.23 when the nanofluid became denser. Therefore, they suggested designing solar thermal collectors containing suitable graphene concentration working fluids to achieve high efficiency.

Vakili et al. [81] examined the effect of weight percent of graphene nanoplatelets on the optical and thermal conductivity properties of the working fluid in DASC application. Their findings were consistent with other works. An increase in graphene nanoplatelets weight percent would result in the improvement of both the absorption coefficient and conductivity of the nanofluid. Particularly, the highest performance of nanofluids was achieved at 0.005 wt% graphene nanoplatelets. This was reflected in the fact that 100% of solar energy was completely absorbed. Another advantage of increasing graphene nanoplatelet concentration was minimizing the collector height. The height of direct absorption collectors ware 2 cm and 10 cm in the cases of 0.005 and 0.00025 wt% graphene nanoplatelet.

Khosrojerdi et al. [191] employed other carbon types to prepare nanofluids to investigate as the working fluid for low-temperature DASCs in the sunlight range from 200 to 2500 nm. The material used in their work was graphene oxide nanoplatelets. The results showed that sunlight energy in the range of 280 to 350 nm was completely absorbed by the nanofluid containing 0.045 wt% of graphene oxide nanoplatelets and 3 cm height of the fluid layer. The results also confirmed that the thermal conductivity coefficient would be changed linearly with nanoparticle weight percentage.

Rose et al. [192] conducted experimental investigations in the absorption of graphene oxide platelets suspended in ethylene glycol for solar radiation (380–800 nm) to qualify a wave optics module. They reported that when the volume fraction of graphene oxide increased, the absorbance proportionally increased. A minimum reflectance and highest absorbance over the visible spectral range could be achieved with an optimum volume fraction of 0.012% of graphene oxide. The experimental results showed a good match with the prediction of the wave optics module. Vallejo et al. [193] investigated the optical properties of water-based nanofluids containing polycarboxylate chemically modified graphene nanoplatelets (P-GNPts) and sulfonic acid-functionalized graphene nanoplatelets (S-GNPts) with concentrations ranging from 0.005 wt% to 0.05 wt%. The prepared nanofluids had moderate stability and good sunlight absorption.

### 4.3. Hybrid Carbon Nanomaterial-Based Nanofluids

Recent studies have indicated that the thermal-optical properties of hybrid carbon nanomaterials are significantly improved in comparison to that of each element. This has made the application of hybrid carbon nanomaterial-based nanofluids for direct thermal solar absorption a promising topic.

The heat transfer properties of carbon materials could be considerably enhanced by introducing nitrogen atoms into their structure. Therefore, Shende and Sundara [107] synthesized nitrogen-doped carbon nanotubes and reduced graphene oxide (N-(rGOMWNTs)) and dispersed different specific amounts of N-(rGOMWNTs) in two types of based solution, including DI water and EG, for comparison. To support the dispersion of carbon materials, a surfactant mixture of polyethylene glycol (PEG) and sodium lauryl sulfate (SLS) with ratio of 2:1 was used. The hybrid structure exhibited an outstanding homogeneity and stability. In a comparison, they found that the nitrogen-doped hybrid carbon-based composite exhibited higher thermal conductivity compared to pure rGO and MWNTs. They demonstrated that the thermal and optical properties of base fluids were improved by adding an even low nanoparticles concentration. The absorption increased and transmittance decreased with the increase in concentration. Moreover, nanofluids using water as the based solution had better optical absorption properties than EG-based one. In detail, the ability to absorb light of DI water-based nanofluids at 50 °C was significantly improved up to 17.7% by adding 0.02% volume fraction of N-(rGOMWNTs). This value was only 15.1% in the case of EG-based nanofluids with even higher volume fractions (0.03%). With the results obtained from experiments, they concluded that nanofluids containing N-(rGO-MWNTs) were considered as an excellent working fluid in solar collector systems.

Another novel method to create a new carbon-based material with good solar absorption properties is to decorate core-shell nanoparticles on the surface of carbon materials, as reported by Fan et al. [194]. They decorated Sn@SiO_2_@Ag onto graphene sheets and dispersed it into DI water to make the working fluid for volumetric solar absorbers. The experimental results exhibited that the decoration of Sn@SiO_2_@Ag onto graphene lead to a great improvement in the plasmon resonance absorption and thermal conductivity of nanofluids. This kind of nanofluids exhibited a strong absorption band in the wavelength range of 250–300 nm and 380–600 nm and the solar absorption coefficient was 2.9 times higher than that of graphene nanofluids. The largest enhancement in thermal conductivity in their study was 16% with 0.3 g/L graphene-coated Sn@SiO2@Ag in nanofluids at 50 °C. This research demonstrated that synthesizing hybrid carbon nanomaterials would be a promising strategy to enhance the performance of working fluids in a volumetric solar collector.

**Figure 9:**
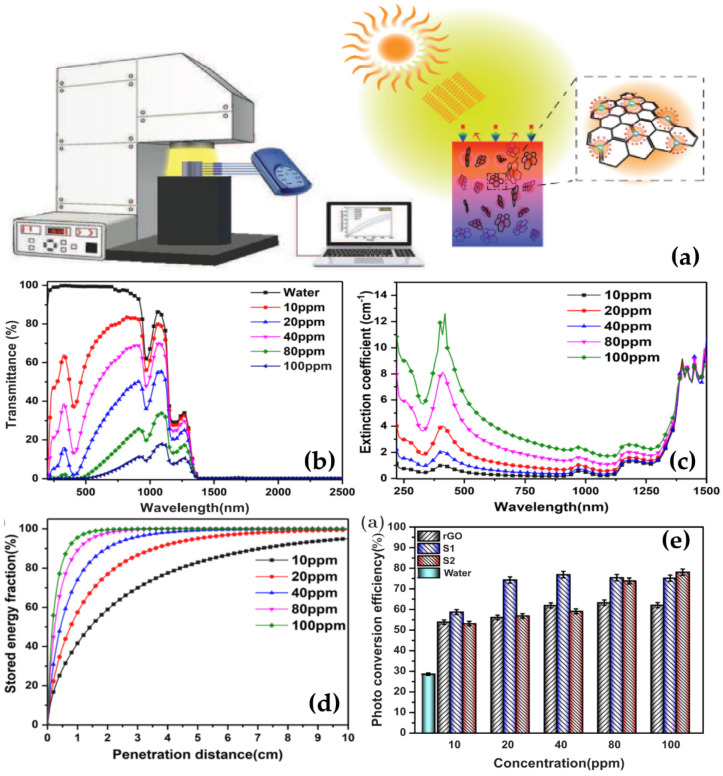
(**a**) Photothermal conversion experiment system; (**b**) transmittance spectra; (**c**) spectral extinction coefficient; (**d**) solar energy absorption fraction as a function of penetration distance of nanofluid containing Ag-rGO for different nanoparticle concentrations; (**e**) collector efficiencies of water and prepared nanofluids containing rGO, Ag-rGO (S1, S2) at different concentration after light irradiation of 2000s [195]. (Reproduced with permission from Elsevier).

Recently, Mehrali et al. [195] employed silver nanoparticles, another great thermally conductive nanoparticle, and graphene to prepare a hybrid material through the straightforward wet-chemical method. This material was applied for producing working fluids in volumetric solar absorbers. The sun irradiance was simulated by an artificial sunlight simulator (WACOM-WXS-90S), as shown in Figure 9. Quartz cuvettes with a 10 mm beam path length were used to examine the optical transmittance spectra (wavelength: 200–2500 nm) of the nanofluids at room temperature. The results indicated that the average transmittance of the Ag-rGO nanofluids (76% for 10 ppm and below 5% for 100 ppm with the nanofluid prepared from 0.3 g of GO and 0.15 g of AgNO_3_) was lower than that of the rGO nanofluids (80% for 10 ppm and 10% for 100 ppm with the nanofluid prepared from 0.3 g of GO). This meant that the Ag-rGO nanofluids had a better average absorption ability compared to rGO nanofluids. They also reported that the Ag decoration improved the thermal conductivity of the rGO nanofluids because Ag nanoparticles decorated on the graphene edges played the role of a thermal bridge between the nanosheets and the bulk fluid. Thanks to the presence of 40 ppm of the hybrid Ag-rGO nanosheets, a great enhancement (2.7 times) of the collector efficiency (77%) was obtained. They made a realistic collector with a height of 20 mm for practical applications.

**Figure 10:**
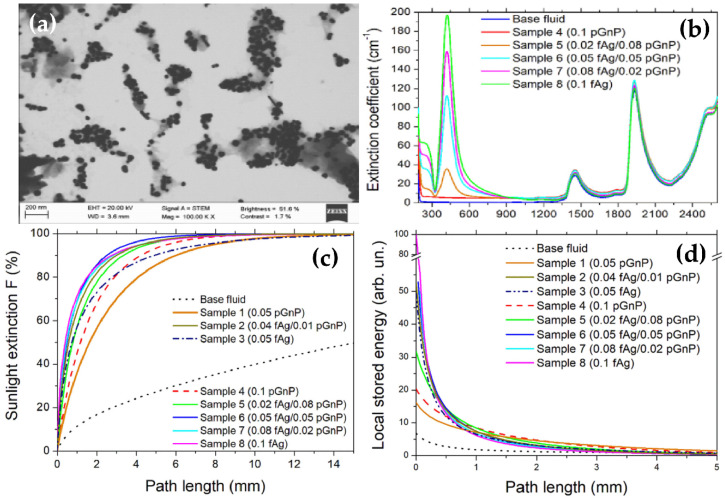
(**a**) TEM images of the dried (0.08 wt% fAg/0.02 wt% pGnP) hybrid nanopowder; (**b**) spectral extinction coefficients for nanofluids containing single and dual nanoadditives. (**a**) 0.05 wt% total concentrations; (**b**) 0.1 wt% total concentration. (**c**) Sunlight extinction for all samples; (**d**) comparison of stored energy distributions for all samples [196].

Vallejo et al. [196] prepared a new type of nanofluid using propylene glycol:water 10:90 wt% mixture as a base fluid and containing hybrid nanoadditive (Ag/GNP) with different concentrations. The obtained results indicated that the nanofluids have good stability via dynamic light scattering measurements. Besides, the extinction coefficient spectra could be modified by adding hybrid nanoadditives with more than 90% sunlight extinction achieved for a path length lower or equal to 6 mm. The shortest sunlight penetration was obtained with hybrid samples (ratios 1:1 and 4:1) for both 0.05 and 0.1 wt% total nanoadditive concentrations (Figure 10). Sani et al. [197] investigated the nanofluids contained low concentrations of sulfonic acid-functionalized GnP and polycarboxylate chemically modified GnP for solar applications. The obtained results indicated that the spectral extinction coefficient increased with respect to water, nanoadditive and larger effect for P-GnP. The P- and S-GnP dispersions containing 0.05% nanoadditive concentration show an extinction of sunlight completed after 5 mm and 25 mm, respectively. Besides, an optically non-linear behavior at high input intensities is observed for both nanoadditive types.

### 4.4. Other Carbon Nanomaterial-Based Nanofluids

The performance of nanofluids containing other carbon nanomaterials, such as carbon nanospheres, graphite, carbon black, diamond nanoparticles, in direct solar energy absorption has been also recently investigated.

Sani et al. [37] improved the overall efficiency of direct sunlight absorbers by nanofluids containing single-walled carbon nanohorns (SWCNHs). They found that almost 100% of the solar spectrum was absorbed in the first centimeter of penetration depth with the concentration of 0.05 g/L, whereas for the lower nanoparticle fraction only 80% of energy was extinct after a 10 cm depth inside the nanofluid. In addition, thermal conductivity was enhanced by up to 10% at the investigated concentrations compared to pure water. Furthermore, carbon nanohorn nanofluids were investigated in the studies of Mercatelli et al. [88], Sani et al. [41], and Moradi et al. [198]. The results of their studies indicated that the nanofluids containing carbon nanohorns could be positive for improving the efficiency of direct thermal solar absorption devices. Poinern et al. [74] has studied the photothermal properties of the nanofluids consisting of carbon nanospheres (CNS) dispersed in Milli-Q^®^ water as working fluids of direct solar absorption collectors. The results showed that the addition of CNS in the base solution could greatly improve the photothermal response. The largest CNS mass content (0.04 g) nanofluid had the largest temperature enhancement of 8.1 °C. Karami et al. [102] reported the results of their studies on carbon nanoball nanofluids. The extinction coefficient of nanofluid-based water and EG containing 300 ppm carbon nanoballs was about 3.9 cm^−1^ and 3.4 cm^−1^, respectively, which were higher compared to the base fluids. The measured spectral transmission demonstrated that the presence of carbon nanoballs leads to raising the optical properties of the nanofluids even at low concentrations. Ladjevardi et al. [97] studied on using nanofluids containing graphite as heat transfer media for direct solar energy absorption. They found that nanofluids containing around 0.000025% graphite in volume fraction can absorb more than 50% of incident irradiation energy, which is much higher compared to pure water solar collector (around 27%). The cost of preparing graphite nanofluids slightly increased (just about 0.0045 $/L). Han et al. [40] prepared carbon black nanofluids to apply for solar absorption. The obtained results revealed that the nanofluids had high thermal conductivity and good absorption in the wavelength range from 200 to 2500 nm. Zeiny et al. [199] compared the photothermal conversion efficiency of carbon black nanofluids to gold, copper and their hybrids. They confirmed that the nanofluids containing nanoparticles could enhance the solar photothermal conversion efficiency, in which carbon black nanofluids showed the highest improvement compared to other nanofluids. This is attributed to their broad and high absorbance spectrum. Considering both photothermal conversion efficiency and potential economic cost, carbon black nanofluid could be considered as the most suitable candidate for direct solar absorption in this study. Sani et al. [104] prepared graphite/diamond ethylene glycol nanofluids and estimated their potential for direct absorption solar collectors. They reported that 100% sunlight extinction could be achieved because of high extinction coefficient, low transmittance and high sunlight absorption of the nanofluids. Gimeno-Furio et al. [200,201] prepared the thermal oil-based nanofluids containing carbon nanoparticles. The experimental results confirmed that carbon nanoparticles are one of the promising candidates for the development of nanofluids in conventional solar collectors due to high solar absorption, cheap and excellent optical properties. The same research team has developed the new deionized water-based nanofluids containing CNHs with an average diameter of 55 ± 15 nm. The nanofluids had good stability even at high temperature by employing a new strategy called double stabilization. This approach consisted of adding a second surfactant that provided stability to the nanofluids even at medium-high temperatures. In addition, they demonstrated that the absorption spectra the nanofluid with double stabilization is increased after exposing to the artificial sun absorption and better than one with single stabilization which contained only one surfactant [202].

## 5. Challenges and Recommendations for Future Work

### 5.1. Challenges

The application of carbon nanomaterial-based nanofluids for direct thermal solar absorption has some possible challenges that have hindered the development of this field. However, most studies focus only on the thermal properties of the nanofluids without mentioning these difficulties. Therefore, this paper briefly presents several important issues that should be carefully investigated in the near future.

#### 5.1.1. Instability of Nanoparticles Dispersion

In order to reach the maximum efficiency of application of carbon nanomaterial-based nanofluids for direct thermal solar absorption, it is necessary to keep the suspension of nanoparticles in the nanofluids. If agglomeration occurs, the concentration of nanoparticles will significantly decrease. This results in a reduction of the ability of the nanofluids to absorb and transfer heat. In addition, sedimentation hinders the process of fluid circulation. It should be noted that the agglomerations tent to be more serious at high temperatures. Generally, the most effective solution to stabilize the nanoparticles is to use techniques such as ultrasonication, mechanical stirring, and surfactants when preparing the nanofluids. However, stabilizing agents can change the optical, thermal properties of the carbon nanoparticles and influence the efficiency of the nanofluids [71,144]. Therefore, stability is one of the most pressing issues that needs to be addressed for high performance carbon nanomaterial-based nanofluids.

#### 5.1.2. High Cost

Economic efficiency should be considered by the high production cost. Difficulties in preparing nanoparticles with a good size and high purity, dispersing nanoparticles into a base fluid, and stabilizing suspension have contributed to the high cost of the carbon nanomaterial-based nanofluids.

#### 5.1.3. Pump Power and Pressure Loss

The solar adsorption ability of the carbon nanomaterial-based nanofluids is enhanced when the volume fraction of the nanoparticles increases. However, increasing the nanoparticle concentration results in high viscosity and flow resisting force [40], which are closely associated with the pump power and pressure loss. Therefore, choosing the optimum nanoparticle concentration is challenging, with a tradeoff between both thermal conductivity and viscosity enhancement.

#### 5.1.4. Erosion of Components

The existence of the carbon nanoparticles not only increases the viscosity of the nanofluids but may also lead to erosion of components in the direct thermal solar absorption systems, such as pumps and pipes. This challenge has contributed to the disadvantages of employing carbon nanomaterial-based nanofluids as a working fluid to absorb and transfer heat.

### 5.2. Recommendations for Future Work

The current results indicate that carbon nanomaterial-based nanofluids are potential candidates for direct thermal solar absorption by their outstanding thermal properties. To develop this work comprehensively, however, future research needs to focus on overcoming the challenges mentions in the previous section. An examination of the influence of the carbon particle shape and size on the efficiency of the thermal solar absorption is also necessary because no current experimental studies have been conducted to gain a better understanding about it. In addition, we would like to recommend using hybrid carbon nanomaterials to synthesize working fluids for direct absorption solar thermal collectors. The thermal properties of the hybrid carbon nanofluids are greater than that of carbon nanofluids, as reported in the studies of Yarmand et al. [203], Sajid and Ali [204], Han et al. [205], Devarajan et al. [206], Baby and Ramaprabhu [207], and Sundar et al. [208].

## 6. Conclusions

This paper provides an overview of using carbon nanomaterial-based nanofluids for direct thermal solar absorption. The recent studies have shown that they can significantly enhance the thermal conductivity of working fluids and the efficiency of direct solar energy systems. The enhancement depends on various factors, such as the type of nanoparticle suspension and the base fluid, the particle volume fraction, the size and shape of the nanoparticles, and the temperature. Theoretically, carbon nanomaterial-based nanofluids are potential candidates for replacing conventional working fluids but there are some challenges that have hindered the development of this field, such as instability of nanofluids, the effect of steam generation, the high cost, the increased pumping power, and the erosion and corrosion of the heat transfer equipment. Therefore, overcoming these issues is necessary. Furthermore, due to high thermal conductivity, using hybrid carbon nanomaterials as nanoparticles to improve the thermal properties of working fluids in direct thermal solar systems should be considered.

## Figures and Tables

**Table 1:** Summary of modeling studies on the optical properties of nanofluids.

Ref.	Type of Work	Particle Type, Diameter-Length	Base Fluid	Model	Remark
[95]	Modeling + experimental	MWCNTs 30 nm–4 µm	Water	Lambert–Beer	Calculated penetration depth on the absorbed sunlight fraction.
[96]	Modeling + experimental	CNTs, 2 nm–10 µm; CNP, 6 nm	Ethanol	Rayleigh approximation	Predict extinction coefficients. A qualitative agreement in visible, disagreement in the UV range.
[97]	Modelling	Graphite, 50 nm	Water	Rayleigh approximation	Calculated the extinction coefficients, studied the effect of volume fractions and diameter variations.
[7]	Modeling + experimental	Graphite, Cu, Al, TiO_2_, 30 nm	Water/VP-1 oil	Maxwell–Garnett and Rayleigh approximation	The approximation works well with water-based nanofluids containing graphite nanoparticles but less well with metallic nanoparticles and/or oil-based fluids
[94]	Modeling + experimental	MWCNT, 53 nm–0.5 µm	Water	Maxwell–Garnett and Rayleigh approximation	Maxwell–Garnett model, which can consider the shape effect; Rayleigh scattering approximation, which can consider size effect.
[31]	Modelling	GNPs	Water	Rayleigh approximation and Lambert–Beer	Investigated the effect of heat losses, solar concentration, nanoparticles loading and channel height on the efficiency of a volumetric flow receiver.
[100]	Modeling + experimental	MWCNT, 10 nm–10 µm	Water	Rayleigh approximation	Investigated the thermal performance of a low-temperature flat plate DASC

**Table 2:** Summary of experimental results on optical properties of nanofluids.

Ref.	Particle Type	Base Fluid	Particle Concentration	Remark
[101]	MWCNT	Water	0.0015–0.01 wt%	0.01 wt% MWCNT-H_2_O nanofluid, the transmittance was nearly zero.
[94]	MWCNT	Water	0.0005 vol%	Solar energy can be completely absorbed in the penetration depth of 10 cm.
[82]	MWCNT	Water	0.0005–0.002 vol%	The extinction coefficient strongly depends on the surfactant.
[103]	SWCNT	Water	5–100 mg/L	Functionalized SWCNT dispersions show less temperature sensitivity by increasing temperature. Maximum absorption occurs at 60 mg/L concentration.
[77]	GO	Water	0.0001–0.1 wt%	Reduced transmittance in the wavelength range from 220 nm to 2000 nm. The optical absorption property of the nanofluids increases with the mass fraction of GO.
[105]	GO and GO/silver hybrid	Water	100 ppm	GO/silver hybrid nanofluids showed the best behaviors under low and high solar radiation.
[73]	MWCNT	EG	0.002 vol%	The average transmittance is about 45% lower than that of pure EG.
[76]	PUMWNT	EG	0.005–0.03 vol%	PUMWNTs nanofluids have lower transmittance than pure EG from 200 to 1400 nm.
[104]	Graphite/nanodiamond	EG	0.0025–0.01 wt%	Demonstrated the generation of vapor bubbles around nanoparticles at light intensities.
[102]	CNB	EG/Water	0.03 wt%	The extinction coefficient of pure water and ethylene glycol is increased by about 3.9 cm^−1^ and 3.4 cm^−1^.

**Table 3:** Summary of experimental results of thermal conductivity measurement of graphene-based nanofluids.

Particle Type	Base Fluid	Particle Concentration	Temperatures	Enhancement	Ref.
Functionalized graphene	Water	0.1–1 wt%	5–35 °C	11.9% to 22.2%	[111]
Functionalized graphene	Water	0.05–1 wt%	25–65 °C	Up to 100%	[118]
Graphite oxide	Water	0.05–0.25 wt%	10–40 °C	14.75–47.57%	[126]
Functionalized graphene	Water	0.05, 0.15, 0.25 wt%	20–60 °C	24.4–33.9%	[112]
SnO_2_/reduced graphene	Water	0.01–0.05 wt%	10–50 °C	3.8–17%	[127]
Highly crumpled few layer graphene	Water	0.001–0.01 wt%	20–50 °C	10–43%	[122]
Graphene quantum dots	Water	0.001–0.002 wt%	20–50 °C	5–18%	[123]
Copper oxide-decorated graphene	Water	0.005–0.05 wt%	25–50 °C	23–90%	[124]
Silver-decorated graphene	Water	0.005–0.05 wt%	25–70 °C	7–86%	[125]
Mono-layer graphene	Water	0.001–0.01 wt%	20–50 °C	8–26%	[128]
Functionalized graphene	Water	0.05, 0.1, 0.15 vol%	10–50 °C	37.2%	[114]
Functionalized graphene	Water	0.005–0.056 vol%	20–50 °C	14–64%	[108]
Nitrogen doped graphene	Water	0.005–0.02 vol%	25–50 °C	Up to 17.7%	[107]
Functionalized graphene	EG	1–5 vol%	10–60 °C	10.5–61%	[106]
Graphene nanoplatelets	EG	0.5–4 vol%	10–90 °C	Up to 32%	[120]
Functionalized graphene	EG	0.041–0.395 vol%	10–70 °C	15–100%	[117]
Functionalized Gr nanoplatelet	PG:Water 30:70 wt%	0.25–1 wt%	20–50 °C	4.7–16%	[115]
Few-layer graphene	Polymer	0.55–1 vol%	10–60 °C	18–25%	[119]
Functionalized graphene	Silicon oil	0.01–0.07 wt%	20–60 °C	Up to 18.9%	[121]

**Table 4:** Summary of experimental results of thermal conductivity of CNT-based nanofluids.

Particle Type	Base Fluid	Particle Concentration	Temperatures	Enhancement	Ref.
MWCNT	Water	1.0 wt%	30 °C	20%	[142]
MWCNTs	Water	0.01–0.5 wt%	25 °C	Up to 22.2%	[136]
SWCNT	Water	0.05–0.25 vol%	30–60 °C	2.84–36.39%	[137]
MWCNTs	Water	0.03 vol%	30–70 °C	33%	[132]
SWCNT	EG	1.1 wt%	22 °C	35%	[131]
MWCNTs	EG	0.12–0.4 wt%	25–60 °C	Up to 72%	[138]
MWCNTs	EG	1–2 vol%	Room temperature	12.4–30%	[139]
SWCNT	EG	0.3 vol%	60 °C	16%	[141]
SiO_2_-MWCNTs	EG	0.05–1.95 vol%	30–50 °C	Up to 22%	[140]
MWCNTs	EG	0.03 vol%	30–70 °C	40%	[132]
Gr-MWCNT/Cu	EG	0.005–0.035 vol%	30–60 °C	10–41%	[135]
MWCNTs	Water–EG	0.45 vol%	40 °C	19.75%	[143]
SWCNT	Poly-alpha-olefins	1.1 wt%	22 °C	12%	[131]
MWCNTs	Oil	0.5 vol%	Room temperature	8.7%	[144]

**Table 5:** Summary of selected experimental results on viscosity of nanofluids containing carbon structures.

Particle Type	Base Fluid	Particle Concentration	Temperature	Behavior *	Ref.
Functionalized graphene nanoplatelet	PG:W 30:70 wt%	0.25–1 wt%	20–50 °C	N	[115]
Functionalized GNPs	PG:W 10:90 wt%	0.25, 0.50 wt%	25–50 °C	N	[148]
CNT	water	0.01–0.75 wt%	0–40 °C	N (low concentrations)/n-N (high concentrations)	[149]
MWCNT	water stabilized by cationic chitosan	Up to 3 wt%		N (low concentrations)/n-N (high concentrations)	[150]
MWCNT	water stabilized by Gum Arabic	1 wt%	15 °C, 30 °C	n-N	[142]
MWCNT	water stabilized by Gum Arabic	0.5 wt%	15 °C, 30 °C, 45 °C	n-N	[151]
ND	water	0.25–2 vol%	25 °C	n-N	[152]
ND	EG	Up to 10 wt%	25 °C	n-N	[153]
ND/graphite	EG	1–5 wt%	25 °C	n-N	[155]
Carbon nanohorn	EG	0.1–1.5 vol%	25 °C	N (low concentrations)/n-N (high concentrations)	[156]

* N, Newtonian; n-N, non-Newtonian.

**Table 6:** Summary of selected experimental results on the solar steam generation of nanofluids containing carbon structures.

Particle Type	Particle Concentration	Container	Radiation Source	Efficiency	Ref.
AuNP decorated GO	500 mg/L	Petri dish	Natural sunlight passing through Fresnel lens	η = 59.2%	[171]
Carbon black	3 wt%	Glass tube	Natural sunlight with parabolic solar collector	η = 73%	[172]
Graphitized carbon black (GCB), carbon black (CB), graphene	0.5 wt%	Acrylic cylinder	Solar simulator (10 suns)	η = 67 ± 4% for GCB η = 69 ± 4% for CB η = 68 ± 4% for graphene	[169]
SWCNTs	2.38 × 10^−4^–19.04 × 10^−4^ vol%	Acrylic tube	Solar simulator (1–10 suns)	η = 45%	[173]
Fe3O4@CNT	1.0 × 10^−2^–6.25 × 10^−2^ g/L	Acrylic beaker	Solar simulator (1–10 suns)	η = 60.32%	[168]
Graphene oxide	0.001–0.004 wt%	Glass beaker	Solar simulator (1600 W xenon lamp; 1.5–3.5 suns	η = 22% and 36.5% at 3.5 and 1.5 suns	[174]
MWCNT-OH	0.002% wt%	Glass beaker	Solar simulator (1.2–3.2 suns)	η = 39%	[167]
rGO, rGO + Ag	1 mg/mL	Acrylic tubes	Solar simulator (1–4 suns)	η = 69% for RGO η = 91.6% for RGO + Ag	[175]

## Abbreviations

CNMs	Carbon nanomaterials
CNSs	Carbon nanospheres
CNTs	Carbon nanotubes
CTAB	Cetyl trimethyl ammonium bromide
D	Diameter
DA	Denatured alcohol
DB	Dodecyl betaine
DI	Deionized
DTAB	Dodecyl trimethyl ammonium bromide
SWCNTs	Single-walled carbon nanotubes
DWCNTs	Double-walled carbon nanotubes
MWCNTs	Multiwalled carbon nanotubes
PUMWCNTs	Partially unzipped multiwalled carbon nanotubes
f-CNTs	Functionalized carbon nanotubes
CNHs	Carbon nanohorns
SWCNHs	Single-walled carbon nanohorns
EG	Ethylene glycol
GNPts	Graphene Nanoplatelets
GNPs	Graphite nanoparticles
GO	Graphene oxide
Gr	Graphene
rGO	Reduced graphene oxide
L	Length
PG	Propylene glycol
PVP	Polyvinyl pyrrolidone
SDBS	Sodium dodecyl benzene sulfonate
SDS	Sodium dodecyl sulfate
SOCT	Sodium octanoate
TH	Therminol
DASCs	Direct absorption solar collectors
(β-CD)	β-cyclodextrin
